# Impact of lenalidomide-bortezomib-dexamethasone induction on patients with newly diagnosed multiple myeloma and renal impairment: Results from the Connect® MM Registry

**DOI:** 10.1038/s41408-024-01177-6

**Published:** 2024-11-11

**Authors:** Sikander Ailawadhi, Hans C. Lee, James Omel, Kathleen Toomey, James W. Hardin, Cristina J. Gasparetto, Sundar Jagannath, Robert M. Rifkin, Brian G. M. Durie, Mohit Narang, Howard R. Terebelo, Prashant Joshi, Ying-Ming Jou, Jorge Mouro, Edward Yu, Rafat Abonour

**Affiliations:** 1 Mayo Clinic, Jacksonville, FL USA; 2 The University of Texas MD Anderson Cancer Center, Houston, TX USA; 3 Myeloma Research Advocate/Advisor, Grand Island, NE USA; 4 Steeplechase Cancer Center, Somerville, NJ USA; 5 University of South Carolina, Columbia, SC USA; 6 Duke University Medical Center, Durham, NC USA; 7 Mount Sinai Hospital, New York, NY USA; 8 Rocky Mountain Cancer Centers US Oncology, Denver, CO USA; 9 Cedars-Sinai Medical Center, Los Angeles, CA USA; 10 US Oncology Research, Columbia, MD USA; 11 Providence Cancer Institute, Southfield, MI USA; 12 Bristol Myers Squibb, Princeton, NJ USA; 13 Indiana University, Indianapolis, IN USA

**Keywords:** Myeloma, Medical research

## Abstract

Limited data exist on the effects of induction treatment in patients with newly diagnosed multiple myeloma (NDMM) and renal impairment (RI), who may also be ineligible for autologous stem cell transplant. This analysis investigated the impact of lenalidomide-bortezomib-dexamethasone (RVd) induction on renal function in patients from the Connect® MM Registry based on transplant status. Eligible patients were aged ≥18 years with symptomatic MM diagnosed ≤2 months before enrollment. Patients in this analysis received front-line RVd for ≥3 cycles and were grouped by transplant status and baseline renal function. As of August 4, 2021, 344 transplanted and 289 non-transplanted patients had received RVd for ≥3 cycles at induction. Improved renal function was observed at 3, 6, and 12 months in patients with all severities of RI at baseline. In patients with >60 and ≤60 creatinine clearance mL/min at baseline, median progression-free survival was 49.4 months and 47.6 months in transplanted patients and 35.7 months and 29.1 months in non-transplanted patients, respectively. These results provide real-world evidence that patients with NDMM and RI who receive front-line RVd for ≥3 cycles may have improved renal function regardless of transplant status, with renal function no longer affecting the long-term outcome. Clinical trial information: NCT01081028.

## Introduction

Renal impairment (RI) is one of the defining events of multiple myeloma (MM) and is present in up to 50% of patients at the time of diagnosis [1, 2]. The International Myeloma Working Group (IMWG) defines RI in MM as serum creatinine levels of >2 mg/dL (>177 µmol/L) or reduced creatinine clearance (CrCl) of <40 mL/min, either or both of which are attributable to the underlying plasma cell disorder [3]. In MM, RI often worsens with progression of disease [4], with recovery less likely to occur with advanced disease than with newly diagnosed MM (NDMM) [5]. In addition, RI is associated with reduced overall survival (OS) and increased risk of early mortality [4]; a pooled analysis from randomized trials showed that the relative risk of disease progression or death was higher in patients with NDMM and RI than those without [6]. Furthermore, RI represents a negative prognostic factor in MM in the first 6 months after diagnosis, with renal recovery being one of the strongest markers associated with patient survival [7]. Renal function improvement, especially when it occurs early (eg, within 1–2 months of therapy initiation), is associated with longer OS and improvement in certain aspects of patient quality of life (QoL) [8, 9]. Thus, RI in MM should be appropriately treated to improve long-term survival outcomes and patient QoL. Patients with MM who present with RI, however, are often excluded from randomized trials due to concerns of potential nephrotoxicity, leading to inconsistent reporting and a lack of data regarding treatment of patients with MM and RI [6, 10, 11]. Lenalidomide, an agent commonly used as part of frontline therapy for MM, is predominantly excreted by the kidney, leading to an increased risk of toxic reactions for patients with RI; it is also known to have kidney-related adverse effects such as dysuria, renal failure, hematuria, acute renal failure, azotemia, calculus ureteric, and renal mass, and dose adjustments are recommended based on the severity of RI [12, 13]. The lack of data in this population is also complicated by the potential ineligibility of these patients to receive autologous stem cell transplant (SCT) [14].

The Connect® MM Registry (NCT01081028) is a large, United States (US)-based, multicenter, prospective observational cohort study designed to examine real‑world diagnostic and treatment patterns [15, 16], clinical outcomes [17], and patient-reported outcomes on health-related QoL [18] in patients with NDMM. Through this noninterventional registry, real-world outcomes and characteristics of patients with NDMM have been described, including the effects of lenalidomide-bortezomib-dexamethasone (RVd) induction therapy [19]. RVd has been shown to improve outcomes compared with doublet Rd therapy in the frontline setting [10] and is one of the most common regimens given to patients with NDMM in the US, often considered the preferred regimen regardless of transplant eligibility [20–23]. Furthermore, bortezomib-based regimens have been traditionally preferred in MM and RI due to maintained efficacy across different severities of RI, with similar adverse event profiles and no need for modification of the dose or dosing schedule [24]. In the Connect MM Registry, renal data at baseline and during follow-up were collected, allowing the tracking of renal function over time in a real-world setting. The present analysis aimed to investigate the impact of RVd induction on renal function and renal recovery by transplant status in patients with NDMM from the Connect MM Registry.

## Methods

### Study design and patients

**Fig. 1:**
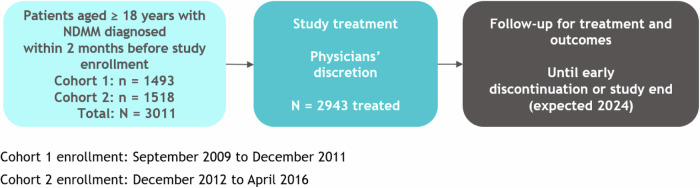
Connect MM registry design. Registered at ClinicalTrials.gov as NCT01081028. NDMM, newly diagnosed multiple myeloma. Data cutoff date: July 23, 2019.

The Connect MM Registry, which has been described previously in detail [15], was designed to characterize treatment patterns and outcomes among patients with NDMM. Eligible patients were aged ≥18 years and diagnosed with symptomatic MM within 2 months prior to enrollment, as defined by the IMWG criteria [25]. Patients from 250 community, academic, and government sites in the US were enrolled into 2 cohorts; patients in cohort 1 (n = 1493) were enrolled from September 2009 to December 2011 and patients in cohort 2 (n = 1518) were enrolled from December 2012 to April 2016 (Fig. 1). Participation in the Connect MM Registry was voluntary, and patients could withdraw at any time without affecting their ongoing medical care. Patients were treated in accordance with standard clinical practice at the discretion of the treating clinician at each site and were followed for treatment and outcomes until early study discontinuation (due to patient withdrawal or death) or end of study.

### Ethics approval and consent to participate

The Connect MM Registry was approved by a central institutional review board (Pro00034753; Advarra, Columbia, MD, USA) or the institutional review board at the individual study site, and all methods were performed in accordance with the relevant guidelines and regulations. Informed consent was obtained from all participants in the Connect MM Registry upon enrollment.

### Analysis population and assessments

This analysis was conducted in patients who received RVd induction for ≥3 cycles (28-day cycles); this excluded patients who may have received one cycle of bortezomib or one cycle of cyclophosphamide- bortezomib-dexamethasone (CyBorD) prior to RVd to ensure that the groups with different renal function at baseline were balanced. Demographics, baseline characteristics, and outcomes were assessed by transplant status. Changes in renal function with RVd induction were assessed in patients grouped according to transplant status and renal function at baseline (calculated CrCl <30 [severe], 30‒50 [moderate], >50‒80 [mild], and >80 [normal] mL/min). SCT was defined as having SCT in the first line with the exception of patients who received SCT after first disease progression, allogeneic/unknown transplants, tandem transplants, or consolidation before maintenance; those whose date of death, discontinuation, cutoff, end of maintenance, or first progression was prior to 100 days after autologous SCT; and 5% of maintenance patients with longest durations from SCT to start of maintenance. Calculated CrCl was determined as follows: [calculated CrCl = (140-age)*weight/(CrCl value*72)] for men and [calculated CrCl = 0.85*(140-age)*weight/(CrCl value*72)] for women. To assess change in renal function from baseline, the maximum post-baseline CrCl value 3 months ( ± 4 weeks), 6 months ( ± 4 weeks), and 12 months ( ± 4 weeks) after the informed consent date was used in this analysis. Progression‑free survival (PFS) and OS were estimated using the Kaplan–Meier method from start of first-line treatment until disease progression or death (whichever occurred first), with patients grouped by transplant status and CrCl ( ≤ 60 or >60 mL/min) at baseline. Patients with progressive disease at baseline were excluded from this analysis.

### Statistical analyses

Descriptive statistics were used to summarize baseline patient characteristics. The median and range were calculated for continuous variables and frequency and percentage were calculated for categorical variables. Unadjusted PFS and OS were estimated using the Kaplan–Meier method and patient data were censored at the last assessment before starting a new line of treatment. Statistical approaches for the analysis of categorical renal status included the Bowker test, Cochran-Mantel-Haenszel test of association, and population-averaged regression models estimating using generalized estimating equations (GEE) [26, 27] to adjust for within-person correlation of repeated measures. The Bowker test of symmetry was performed for each post-baseline timepoint to evaluate the shift/improvement of renal status compared to the baseline renal status for those patients who had both baseline and post-baseline data. Symmetry around the main diagonal in the renal status shift table implies no change/improvement in the renal status between baseline and post-baseline timepoint. The Cochran-Mantel-Haenszel test was used to assess the potential monotone correlation between the ordinal RI severity and time points, controlling for patient. That is, each patient is a stratum and the frequency table of renal status by timepoint is formed for each stratum/patient, with the cell frequency being either 0 or 1. As a third approach, a GEE regression model was estimated and assessed for the repeated measures of the ordinal renal function status. Inference of the population-averaged GEE model assumes proportional odds and models the cumulative logits of renal status, with the cumulative logit being the logarithm of the probability of better outcomes compared to poorer outcomes. Statistical analyses were performed with the use of SAS Enterprise Guide, version 7.15. The Connect MM Registry had the ability to query sites for more information on questionable data and multiple imputation methods in the analyses could be used to mitigate the impact of missing data.

## Results

### Patient characteristics

A total of 3011 patients were enrolled in both cohorts of the Connect MM Registry, including 1493 patients in cohort 1 and 1518 patients in cohort 2 (Fig. 1). As of the cutoff date of August 4, 2021, a total of 633 patients had received RVd in the first regimen for ≥3 cycles, of whom 344 had received SCT and 289 had not received an SCT (non-SCT). At baseline, renal function was normal in 52.1% of patients, of whom 64.2% had received SCT and 35.8% had not; 28.6% had mild RI (48.6% transplanted and 51.4% non-transplanted), 13.6% had moderate RI (38.4% transplanted and 61.6% non-transplanted), and 5.7% had severe RI (30.6% transplanted and 69.4% non-transplanted). Overall, patients with normal renal function at baseline were younger than those with mild, moderate, or severe RI (median age, 59 years vs 67 years), and transplanted were younger than non-transplanted patients for each severity of baseline renal function (Table 1). Gender distribution, patients with Eastern Cooperative Oncology Group (ECOG) performance status, and hypercalcemia (defined as calcium ≥11.5 mg/dL) were generally similar between transplanted and non-transplanted patients for each severity of baseline renal function; in both transplanted and non-transplanted groups, increasing severity of baseline RI was associated with decreasing ECOG performance status and increasing frequency of hypercalcemia. The proportion of Black patients increased with increasing severity of RI regardless of whether they received SCT. Proportion of patients with International Staging System (ISS) disease stage I decreased and the proportion of patients with ISS disease stage III increased with increasing severity of baseline renal function. The proportion of those with anemia, defined as hemoglobin <10 g/dL or >2 g/dL less than lower limit of normal, increased with increasing severity of baseline renal impairment for both transplanted and non-transplanted groups; anemia was more common in the transplanted group than non-transplanted group (40.1% vs 51.2%), particularly in those with severe baseline RI (72.7% vs 96.0%). Cytogenetic abnormalities were generally similar between the 2 groups, except, among transplanted patients, t(4;14) was less common in those with mild baseline renal function (2.3% vs 8.6%) and t(11;14) was more common in those with severe baseline renal function (36.4% vs 8.0%) compared with non-transplanted patients.

**Table 1:** Baseline characteristics by transplant status and renal function in patients receiving induction with RVd.

	Transplanted (n = 344)					Non-transplanted (n = 289)				
Baseline renal function	Normal n = 212	Mild n = 88	Moderate n = 33	Severe n = 11	Overall n = 344	Normal n = 118	Mild n = 93	Moderate n = 53	Severe n = 25	Overall n = 289
Median age, years (range)	58 (24.0–73.0)	63 (38.0–75.0)	60 (42.0–75.0)	61 (37.0–70.0)	59 (24.0–75.0)	61 (35.0–80.0)	71 (46.0–90.0)	72 (52.0–88.0)	70 (51.0–89.0)	69 (35.0–90.0)
Male, n (%)	132 (62.3)	50 (56.8)	20 (60.6)	7 (63.6)	209 (60.8)	81 (68.6)	50 (53.8)	26 (49.1)	11 (44.0)	168 (58.1)
Race, n (%)										
White	181 (85.4)	82 (93.2)	28 (84.8)	6 (54.5)	297 (86.3)	98 (83.1)	73 (78.5)	44 (83.0)	18 (72.0)	233 (80.6)
Black	27 (12.7)	6 (6.8)	4 (12.1)	4 (36.4)	41 (11.9)	15 (12.7)	16 (17.2)	6 (11.3)	5 (20.0)	42 (14.5)
Other^a^	4 (1.9)	0	1 (3.0)	1 (9.1)	6 (1.7)	5 (4.2)	4 (4.3)	3 (5.7)	2 (8.0)	14 (4.8)
ECOG PS, n (%)										
0–1	132 (62.3)	52 (59.1)	23 (69.7)	3 (27.3)	210 (61.0)	76 (64.4)	52 (55.9)	26 (49.1)	11 (44.0)	165 (57.1)
2–3	16 (7.5)	4 (4.5)	1 (3.0)	2 (18.2)	23 (6.7)	13 (11.0)	7 (7.5)	4 (7.5)	2 (8.0)	26 (9)
Calculated ISS stage, n (%)										
I	69 (32.5)	23 (26.1)	2 (6.1)	0	94 (27.3)	23 (19.5)	13 (14.0)	5 (9.4)	0	41 (14.2)
II	63 (29.7)	22 (25.0)	8 (24.2)	2 (18.2)	95 (27.6)	41 (34.7)	29 (31.2)	11 (20.8)	3 (12.0)	84 (29.1)
III	28 (13.2)	17 (19.3)	14 (42.4)	7 (63.6)	66 (19.2)	16 (13.6)	23 (24.7)	20 (37.7)	17 (68.0)	76 (26.3)
Calcium ≥ 11.5 mg/dL, n (%)	12 (5.7)	8 (9.1)	5 (15.2)	2 (18.2)	27 (7.8)	6 (5.1)	11 (11.8)	9 (17.0)	4 (16.0)	30 (10.4)
Hb <10 or >2 g/dL less than LLN, n (%)	71 (33.5)	38 (43.2)	21 (63.6)	8 (72.7)	138 (40.1)	50 (42.4)	40 (43.0)	34 (64.2)	24 (96.0)	148 (51.2)
Del(17p), n (%)										
Yes	36 (17.0)	13 (14.8)	1 (3.0)	2 (18.2)	52 (15.1)	13 (11.0)	12 (12.9)	5 (9.4)	2 (8.0)	32 (11.1)
No	139 (65.6)	61 (69.3)	22 (66.7)	6 (54.5)	228 (66.3)	74 (62.7)	57 (61.3)	32 (60.4)	15 (60.0)	178 (61.6)
Data not available	37 (17.5)	14 (15.9)	10 (30.3)	3 (27.3)	64 (18.6)	31 (26.3)	24 (25.8)	16 (30.2)	8 (32.0)	79 (27.3)
+1q21, n (%)										
Yes	12 (5.7)	4 (4.5)	5 (15.2)	2 (18.2)	23 (6.7)	6 (5.1)	9 (9.7)	6 (11.3)	1 (4.0)	22 (7.6)
No	91 (42.9)	42 (47.7)	12 (36.4)	3 (27.3)	148 (43.0)	45 (38.1)	34 (36.6)	22 (41.5)	10 (40.0)	111 (38.4)
Data not available	109 (51.4)	42 (47.7)	16 (48.5)	6 (54.5)	173 (50.3)	67 (56.8)	50 (53.8)	25 (47.2)	14 (56.0)	156 (54.0)
1 P loss, n (%)										
Yes	3 (1.4)	3 (3.4)	0	0	6 (1.7)	1 (0.8)	2 (2.2)	2 (3.8)	1 (4.0)	6 (2.1)
No	14 (6.6)	10 (11.4)	1 (3.0)	1 (9.1)	26 (7.6)	5 (4.2)	7 (7.5)	5 (9.4)	0	17 (5.9)
Data not available	195 (92.0)	75 (85.2)	32 (97.0)	10 (90.9)	312 (90.7)	112 (94.9)	84 (90.3)	46 (86.8)	24 (96.0)	266 (92.0)
t(14;16), n (%)										
Yes	7 (3.3)	4 (4.5)	1 (3.0)	0	12 (3.5)	2 (1.7)	6 (6.5)	4 (7.5)	1 (4.0)	13 (4.5)
No	73 (34.4)	26 (29.5)	3 (9.1)	4 (36.4)	106 (30.8)	28 (23.7)	24 (25.8)	15 (28.3)	5 (20.0)	72 (24.9)
Data not available	82 (38.7)	37 (42.0)	14 (42.4)	5 (45.5)	138 (40.1)	61 (51.7)	42 (45.2)	26 (49.1)	12 (48.0)	141 (48.8)
t(4;14), n (%)										
Yes	17 (8.0)	2 (2.3)	1 (3.0)	0	20 (5.8)	7 (5.9)	8 (8.6)	1 (1.9)	3 (12.0)	19 (6.6)
No	151 (71.2)	64 (72.7)	21 (63.6)	6 (54.5)	242 (70.3)	74 (62.7)	57 (61.3)	36 (67.9)	12 (48.0)	179 (61.9)
Data not available	44 (20.8)	22 (25.0)	11 (33.3)	5 (45.5)	82 (23.8)	37 (31.4)	28 (30.1)	16 (30.2)	10 (40.0)	91 (31.5)
t(11;14), n (%)										
Yes	36 (17.0)	15 (17.0)	4 (12.1)	4 (36.4)	59 (17.2)	13 (11.0)	14 (15.1)	9 (17.0)	2 (8.0)	38 (13.1)
No	101 (47.6)	43 (48.9)	13 (39.4)	2 (18.2)	159 (46.2)	47 (39.8)	34 (36.6)	26 (49.1)	9 (36.0)	116 (40.1)
Data not available	75 (35.4)	30 (34.1)	16 (48.5)	5 (45.5)	126 (36.6)	58 (49.2)	45 (48.4)	18 (34.0)	14 (56.0)	135 (46.7)
Hyperdiploidy, n (%)										
Yes	14 (6.6)	7 (8.0)	1 (3.0)	1 (9.1)	23 (6.7)	6 (5.1)	11 (11.8)	6 (11.3)	0	23 (8.0)
No	129 (60.8)	45 (51.1)	20 (60.6)	4 (36.4)	198 (57.6)	62 (52.5)	43 (46.2)	22 (41.5)	10 (40.0)	137 (47.4)
Data not available	69 (32.5)	36 (40.9)	12 (36.4)	6 (54.5)	123 (35.8)	50 (42.4)	39 (41.9)	25 (47.2)	15 (60.0)	129 (44.6)

*ECOG PS* Eastern Cooperative Oncology Group performance status, *Hb* hemoglobin, *ISS* International Staging System, *LLN* lower limit of normal, *RVd* lenalidomide, bortezomib, and dexamethasone.

^a^Includes American Indian/Alaskan Native, Asian, Pacific Islander, Other, and Not Specified.

Data cutoff date: August 4, 2021.

### Changes from baseline in renal function

**Fig. 2:**
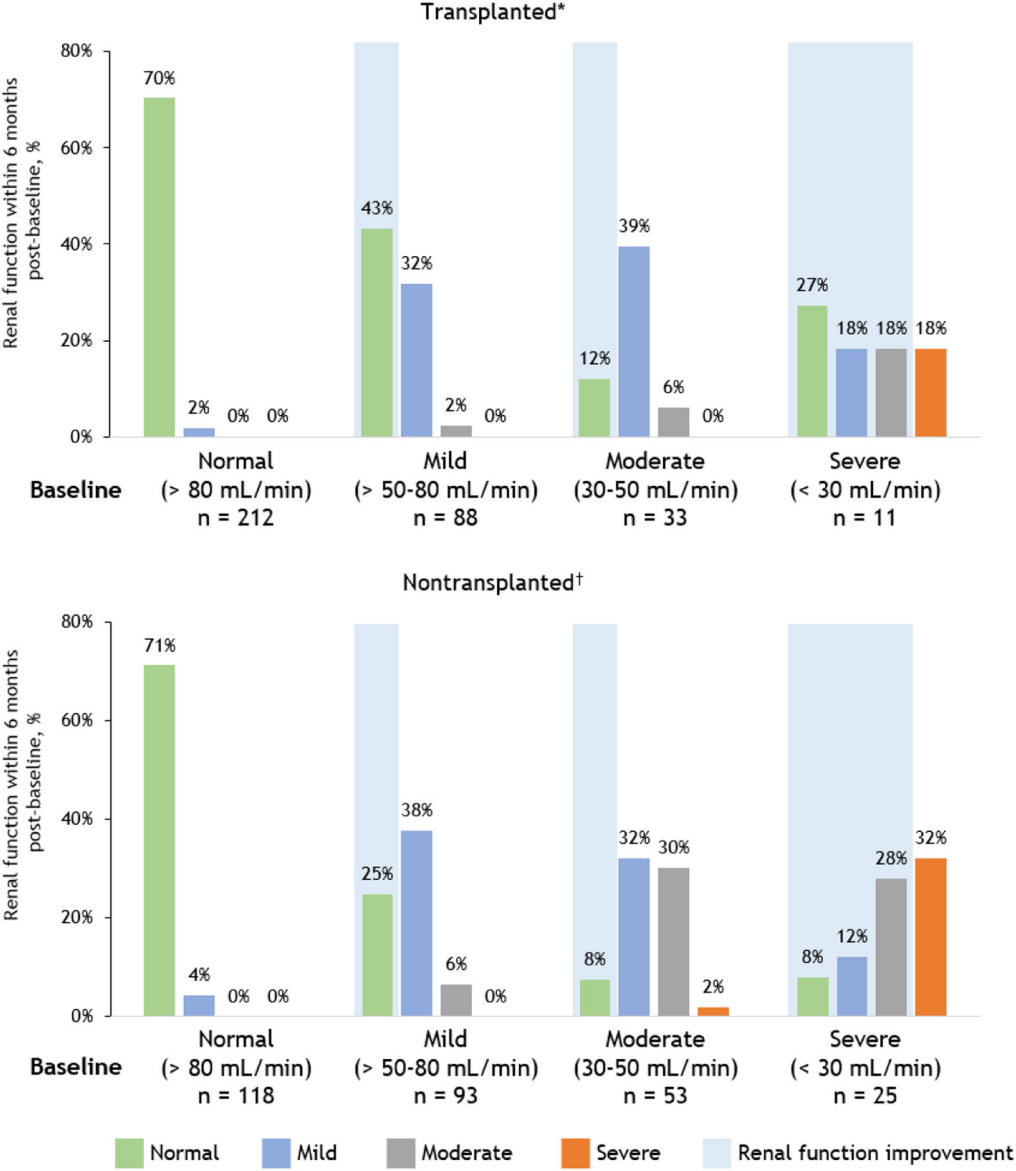
Renal function at 6 months post-baseline by transplant status and baseline renal function. *Missing/death: baseline normal = 28%, baseline mild = 23%, baseline moderate = 42%, baseline severe = 18%. ^†^Missing/death: baseline normal = 25%, baseline mild = 31%, baseline moderate = 28%, baseline severe = 20%. Data cutoff date: August 4, 2021.

Changes in renal function (as measured by CrCl) from baseline to 3-, 6-, and 12-months post-baseline are shown by baseline renal function and transplant status in Figs. 2 and S1. An improvement in renal function refers to an improvement of at least one severity category of renal function. Of the patients with moderate RI at baseline, 63.6%, 51.5%, and 45.4% had an improved renal function in the transplanted group, and 41.5%, 39.6%, and 28.3% had an improved RI in the non-transplanted group at 3-, 6-, and 12-months post-baseline, respectively (Fig. S1). Of the patients with severe RI at baseline, 54.5%, 63.6%, and 54.5% had an improved renal function in the transplanted group and 44.0%, 48.0%, and 36.0% had an improved renal function in the non-transplanted group at 3, 6, and 12 months respectively (Fig. S1). There were 15 deaths, 8 with normal renal function (3 transplanted and 5 non-transplanted), 5 with mild RI (1 transplanted and 4 non-transplanted), 1 with moderate RI (1 non-transplanted), and 1 with severe RI (1 non-transplanted). To summarize, in both transplanted and non-transplanted patients, most patients who were still on the study were observed to have improved or maintained renal function at all measured timepoints, including those with moderate (CrCl 30‒50 mL/min) and severe (CrCl < 30 mL/min) RI at baseline. Based on that, the following statistical analyses were applied to the overall population, combining transplanted and non-transplanted patients.

The Bowker test of symmetry evaluated the shift/improvement of renal status for those patients who had both baseline and post-baseline data (combining the transplant statuses, n = 540, 460, and 417 at 3, 6, and 12 months, respectively, among the 633 patients at baseline). The null hypothesis of the Bowker test: symmetry around the diagonal (i.e., no change in renal status) was rejected given the statistical significance (*P* < 0.0001) for all 3 time points for the overall population, indicating strong statistical evidence of renal function improvement at all 3 time points compared with baseline.

Cochran-Mantel-Haenszel tests for monotone correlation between the ordinal RI severity and (simultaneous) multiple time points, controlling for patient, showed strong statistical evidence of a monotone correlation between RI severity and time points with *P* value < 0.0001 for all types of scores. The result from the GEE models indicated that the odds of better renal status at 3, 6, and 12 months are 1.7 (*P* < 0.0001), 1.9 (*P* < 0.0001), and 1.3 (*P* = 0.0016) times the odds of better renal status at baseline. Thus, with strong statistical evidence, there is post-treatment improvement in renal function, with the best outcome observed at 6 months (Table S1).

### Patient characteristics by change from baseline in renal function

Baseline characteristics of patients with improved or maintained renal function compared with patients with worsened renal function at 12 months are shown in Table 2. At the 12-month time point, 378 patients had improved or maintained renal function and 43 patients had worsened renal function. Overall, patients with improved or maintained renal function were younger than those with worsened renal function (median age, 63.0 vs 67.0 years) and a greater proportion were Black (11.4% vs 4.7%). Calculated ISS stage, ECOG performance status, anemia, and cytogenetic abnormalities were generally similar between the 2 groups.

**Table 2:** Baseline characteristics of patients with improved or maintained renal function compared with patients with worsened renal function at 12 months.

Characteristic	Improved or maintained renal function n = 378	Worsened renal function n = 43
Median age, years (range)	63.0 (27.0‒89.0)	67.0 (46.0‒90.0)
Male, n (%)	224 (59.3)	19 (44.2)
Race, n (%)		
White	322 (85.2)	40 (93.0)
Black	43 (11.4)	2 (4.7)
Other^a^	13 (3.4)	1 (2.3)
ECOG PS, n (%)		
0–1	224 (59.3)	26 (60.5)
2–3	27 (7.1)	2 (4.7)
Calculated ISS stage, n (%)		
I	95 (25.1)	8 (18.6)
II	110 (29.1)	13 (30.2)
III	104 (27.5)	12 (27.9)
Calcium ≥ 11.5 mg/dL, n (%)	37 (9.8)	2 (4.7)
Hb < 10 or >2 g/dL less than LLN, n (%)	167 (44.2)	18 (41.9)
Del(17p), n (%)		
Yes	52 (13.8)	6 (14.0)
No	248 (65.6)	30 (69.8)
Data not available	78 (20.6)	7 (16.3)
1 Q gain, n (%)		
Yes	30 (7.9)	2 (4.7)
No	159 (42.1)	14 (32.6)
Data not available	189 (50.0)	27 (62.8)
1 P loss, n (%)		
Yes	7 (1.9)	0
No	18 (4.8)	3 (7.0)
Data not available	353 (93.4)	40 (93.0)
t(14;16), n (%)		
Yes	14 (3.7)	2 (4.7)
No	117 (31.0)	12 (27.9)
Data not available	159 (42.1)	19 (44.2)
t(4;14), n (%)		
Yes	22 (5.8)	3 (7.0)
No	256 (67.7)	31 (72.1)
Data not available	100 (26.5)	9 (20.9)
t(11;14), n (%)		
Yes	56 (14.8)	7 (16.3)
No	173 (45.8)	19 (44.2)
Data not available	149 (39.4)	17 (39.5)
Hyperdiploidy, n (%)		
Yes	26 (6.9)	0
No	205 (54.2)	27 (62.8)
Data not available	147 (38.9)	16 (37.2)

*ECOG PS* Eastern Cooperative Oncology Group performance status, *Hb* hemoglobin, *ISS* International Staging System, *LLN* lower limit of normal.

^a^Includes American Indian/Alaskan Native, Asian, Pacific Islander, Other, and Not Specified.

Data cutoff date: August 4, 2021.

### Progression-free survival and overall survival

**Fig. 3:**
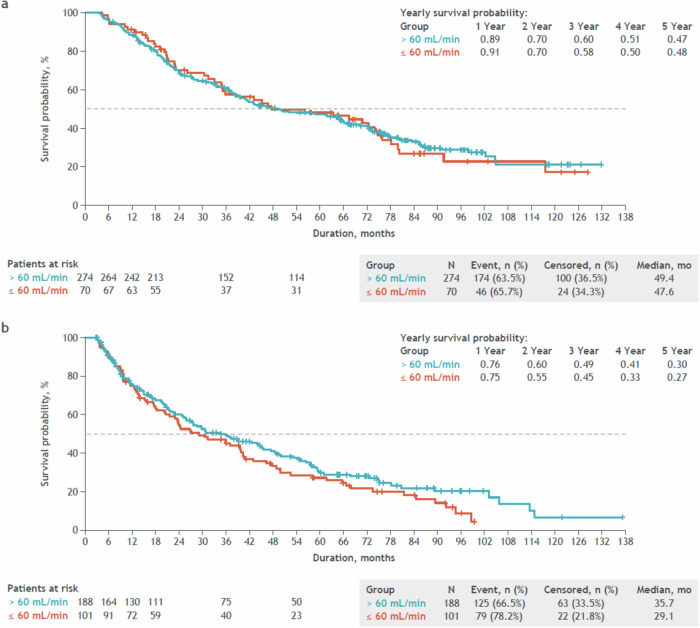
Unadjusted progression-free survival by baseline renal function. **a** Unadjusted progression-free survival by baseline renal function in patients who received a stem cell transplant. **b** Unadjusted progression-free survival by baseline renal function in patients who did not receive a stem cell transplant. Patients with first disease progression date earlier than their informed consent date were excluded from this analysis. Progression-free survival was defined as the time from first dose date to disease progression or death, whichever occurred first. Data cutoff date: August 4, 2021.

Among transplanted patients, the median PFS was 49.4 months in those with >60 mL/min baseline CrCl and 47.6 months in those with ≤60 mL/min baseline CrCl (Fig. 3a). The 5-year PFS rate was 47% for the >60 mL/min group and 48% for the ≤60 mL/min group, indicating that PFS was consistent regardless of baseline renal function among transplanted patients. Among non-transplanted patients, the median PFS was 35.7 months in those with >60 mL/min baseline CrCl and 29.1 months in those with ≤60 mL/min baseline CrCl (Fig. 3b). The 5-year PFS rate was 30% for the >60 mL/min group and 27% for the ≤60 mL/min group, indicating that PFS was generally consistent among non-transplanted patients.

Analysis of PFS was also performed by transplant eligibility, which was reported by each site. Among transplant-eligible patients (n = 421, of whom 344 received actual SCT), the median PFS was 48.8 months in those with >60 mL/min baseline CrCl and 43.2 months in those with ≤60 mL/min baseline CrCl (Fig. S2a). The 5-year PFS rate was 46 and 42%, respectively, indicating that PFS was generally consistent among transplant-eligible patients. Among transplant-noneligible patients, the median PFS was 36.4 months in those with >60 mL/min baseline CrCl and 30.6 months in those with ≤60 mL/min baseline CrCl with a 5-year survival PFS rate of 29% in both groups (Fig. S2b). These results show that similar PFS trends were observed among transplanted and transplant-eligible patients and among non-transplanted and transplant-noneligible patients.

**Fig. 4:**
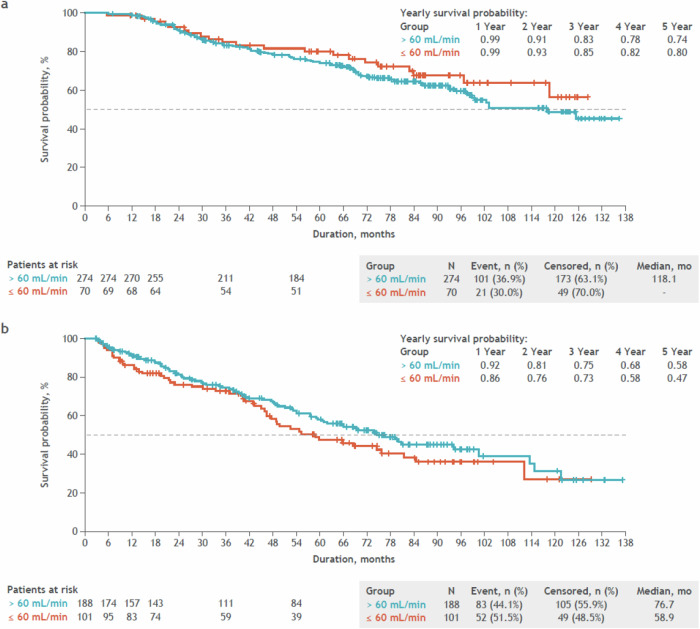
Unadjusted overall survival by baseline renal function. **a** Unadjusted overall survival by baseline renal function in patients who received a stem cell transplant. **b** Unadjusted overall survival by baseline renal function in patients who did not receive a stem cell transplant. Patients with first disease progression date earlier than their informed consent date were excluded from this analysis. Overall survival was defined as the time from first dose date to death. Data cutoff date: August 4, 2021.

The median OS in transplanted patients was 118 months in those with >60 mL/min baseline CrCl and not reached in those with ≤60 mL/min baseline CrCl (Fig. 4a). The 5-year OS rate was 74 and 80%, respectively. The median OS in non-transplanted patients was 76.7 months in those with >60 mL/min baseline CrCl and 58.9 months in those with ≤60 mL/min baseline CrCl (Fig. 4b). The 5-year OS rate was 58% and 47%, respectively.

## Discussion

Renal impairment is considered a poor prognostic factor in MM, and the recovery of renal function is associated with prolonged survival [5, 8]. Because a significant number of patients with MM present with some degree of renal dysfunction at diagnosis, it is important to understand how induction treatment regimens affect renal function recovery as well as patients’ long-term outcomes in routine clinical practice.

In the present analysis of patients with NDMM and RI who received RVd induction for ≥3 cycles, an improvement from baseline in renal function was frequently observed. This improvement, including recovery to normal renal function, was observed at all 3 analysis time points (3, 6, and 12 months) and across various baseline severities of RI regardless of transplant status. Refinement of the renal function assessment window did not alter trends in the improvements in renal function observed. Several formal statistical tests, including Cochran-Mantel-Haenszel tests and the PROC GENMOD procedure for estimating GEE regression models, were utilized, providing strong statistical evidence to support the observed improvements in renal function among patients using RVd at induction. Notably, the 6-month time point was estimated to have the best outcome among the 3 timepoints. Although some baseline characteristics such as ISS stage and race were numerically different between transplanted and non-transplanted groups, improvements in RI were observed across all the groups. Furthermore, baseline clinical characteristics were generally similar between patients who had improved or maintained renal function versus those with worsened renal function from baseline, indicating that RVd resulted in renal improvement regardless of baseline renal function, other clinical characteristics, or transplant status.

Overall, PFS with RVd induction was generally consistent and agnostic to baseline renal function ( ≤60 mL/min or >60 mL/min CrCl), indicating that RVd treatment was associated with improved renal function to a level in which renal function was no longer associated with poor outcomes. This was the case regardless of transplant status or eligibility.

Initial treatment of MM has greatly improved over the past 2 decades, largely due to the availability of effective new therapies such as immunomodulatory drugs (IMiDs), proteasome inhibitors (PIs), and monoclonal antibodies; these agents, typically used in combinations, have significantly prolonged the survival of patients with NDMM [28–31]. However, the risk of early death remains high in patients with severe RI [32]. RVd, or lenalidomide (IMiD) combined with bortezomib (PI) and dexamethasone, is a preferred frontline treatment for patients with NDMM regardless of transplant eligibility [20–23]. Although RVd has been examined in NDMM, patients with comorbidities such as RI and cardiovascular diseases are often excluded from clinical studies [10]; as such, the impact of novel, targeted regimens has not been extensively studied in patients with MM and RI [32] and results from clinical studies may not be representative of real-world practice. One of the reasons why severe RI is an exclusion criterion in clinical trials is due to concerns of potential nephrotoxicity or impaired clearance of the drug [6, 33]. Lenalidomide, for example, is renally excreted and its clearance has been shown to be substantially reduced with increased drug exposure in patients with moderate and severe RI compared with patients with mild RI or normal renal function [34], and the starting dose is recommended to be adjusted according to renal function [35, 36]; thus, it may not always be given as part of frontline treatment for those with moderate and severe RI by physicians. Nonetheless, the present analysis showed that RVd given as induction therapy improves renal function in patients with all levels of RI. This was observed regardless of transplant status. Furthermore, PFS in transplant-eligible patients in this analysis was 48.8 months for those with >60 mL/min CrCl and 43.2 months for those with ≤60 mL/min CrCl, consistent with the phase 3 SWOG S0777 trial, which showed that transplant-eligible patients with NDMM who received RVd had a median PFS of 43 months [10]. In the PrECOG study, which investigated Rd regimen in patients with relapsed MM with impaired renal function, higher doses of lenalidomide could be used safely in patients with renal impairment [13]; patients with CrCl of ≥30 mL/min could receive full dose therapy, similarly to patients with normal renal function. As many patients with NDMM have either normal renal function or mild renal impairment, the absence of requiring dose modifications and the simplification of schedules are highly beneficial for both patients and clinicians.

There are some limitations to this analysis. First, by using 3 cycles of RVd as an inclusion criterion, the time between diagnosis and the receipt of the final cycle of RVd is immortal. However, implementing this criterion was necessary to draw inferences about the targeted population of clinical interest. Second, due to the observational nature of this study, the lack of regularly scheduled clinic visits may have impacted renal assessment measurements and other outcome measures such as PFS, and this may have introduced bias into the analysis. Dose adjustments, which are often necessitated in patients with renal impairment [37] and may have been made based on the physicians’ decision, were also not evaluated in this analysis. Third, the regimens used by patients in the Registry may differ from how patients with NDMM are commonly treated today. The Connect MM Registry was designed prior to the approval of novel therapies and the standard of care and therapeutic landscape have evolved substantially in the timespan between study start and time of manuscript publication. Furthermore, enrollment criteria for various studies in MM have changed with new technology such as testing for high-risk cytogenetics with FISH. Finally, patients treated with one cycle of bortezomib or CyBorD were not included, potentially excluding some patients who had moderate to severe renal dysfunction. Only patients with NDMM who received RVd were included in this analysis to ensure patient groups were as balanced as possible. Despite these limitations, robust, validated data from the Connect MM Registry has resulted in extensive addition to the literature and has been the basis of several real-world analyses and findings for patients with MM. These results from the Connect MM Registry of NDMM patients with RI who received RVd as first-line treatment showed that early improvement in renal function is indeed important for improving long-term outcomes and that appropriate treatment of renal impairment can lead to outcomes similar to those seen in prospective clinical trial settings. These prospective data provide real-world evidence that can be utilized in patient care, and the results presented here show the benefit of evidence-based regimens in real-world patients even with comorbidities like RI. Further prospective research is warranted on RVd induction and other contemporary efficacious combinations in patients with advanced renal disease, including the exploration of newer small-molecule agents, such as novel CELMoD agents (iberdomide and mezigdomide), that are not primarily excreted by the kidneys.

## Supplementary information

Supplement

## Data Availability

Bristol Myers Squibb’s policy on data sharing may be found at https://www.bms.com/researchers-and-partners/clinical-trials-and-research/disclosure-commitment.html.
