# Supplementary material for: Impact of lenalidomide-bortezomib-dexamethasone induction on patients with newly diagnosed multiple myeloma and renal impairment: Results from the Connect® MM Registry
Source: Blood Cancer J. 2024 Nov 11;14(1):198. doi: 10.1038/s41408-024-01177-6 (PMC11554676; doi:10.1038/s41408-024-01177-6)

**Impact of lenalidomide-bortezomib-dexamethasone induction on patients with newly diagnosed multiple myeloma and renal impairment: Results from the Connect® MM Registry**

# **Supplementary information**

## **Table S1. GEE analysis result of longitudinal renal status data.**

ChiSq, Chi square; GEE, generalized estimation equations. *Odds ratio of better outcomes.

|  | | | **Estimate  (95% CI)** | | **Standard error** | | **Z** | | **Pr > \|Z\|** |
| --- | --- | --- | --- | --- | --- | --- | --- | --- | --- |
| **Intercept 1, =clogit of Pr(normal) at BL** | | | 0.0572 (−0.10-0.21) | | 0.0788 | | 0.73 | | 0.4676 |
| **Intercept 2, =clogit of Pr(mild or better) at BL** | | | 1.4975 (1.30-1.69) | | 0.1003 | | 14.93 | | < 0.0001 |
| **Intercept 3, =clogit of Pr(moderate or better) at BL** | | | 2.9613 (2.61-3.31) | | 0.1774 | | 16.70 | | < 0.0001 |
| **Time 3 mo, = log odds ratio of 3 mo vs BL** | | | 0.5515 (0.41-0.69) | | 0.0729 | | 7.56 | | < 0.0001 |
| **Time 6 mo, = log odds ratio of 6 mo vs BL** | | | 0.6478 (0.49-0.80) | | 0.0800 | | 8.10 | | < 0.0001 |
| **Time 12 mo, = log odds ratio of 12 mo vs BL** | | | 0.2500 (0.09-0.41) | | 0.0793 | | 3.15 | | 0.0016 |
|  | **Contrast estimate results** | | | | | | | | |
|  | **Mean estimate  (CI)** | **L’Beta estimate (CI)** | | **Chi-square** | | **Pr > ChiSq** | | **Odds ratio* (CI)** | |
| **3 mo vs baseline** | 0.6345  (0.60-0.67) | 0.5515 (0.41-0.69) | | 57.23 | | < 0.0001 | | 1.7358  (1.50-2.00) | |
| **6 mo vs baseline** | 0.6565  (0.62-0.69) | 0.6478  (0.49-0.80) | | 65.60 | | < 0.0001 | | 1.9113 (1.63-2.24) | |
| **12 mo vs baseline** | 0.5622  (0.52-0.60) | 0.2500 (0.09-0.41) | | 9.93 | | 0.0016 | | 1.2840  (1.10-1.50) | |

## **Figure S1. Renal function at 3, 6, and 12 months post-baseline by transplant status and baseline renal function.**

*Death indicates a patient had missing creatinine clearance data and died ± 4 weeks of each time point. Data cutoff date: August 4, 2021.


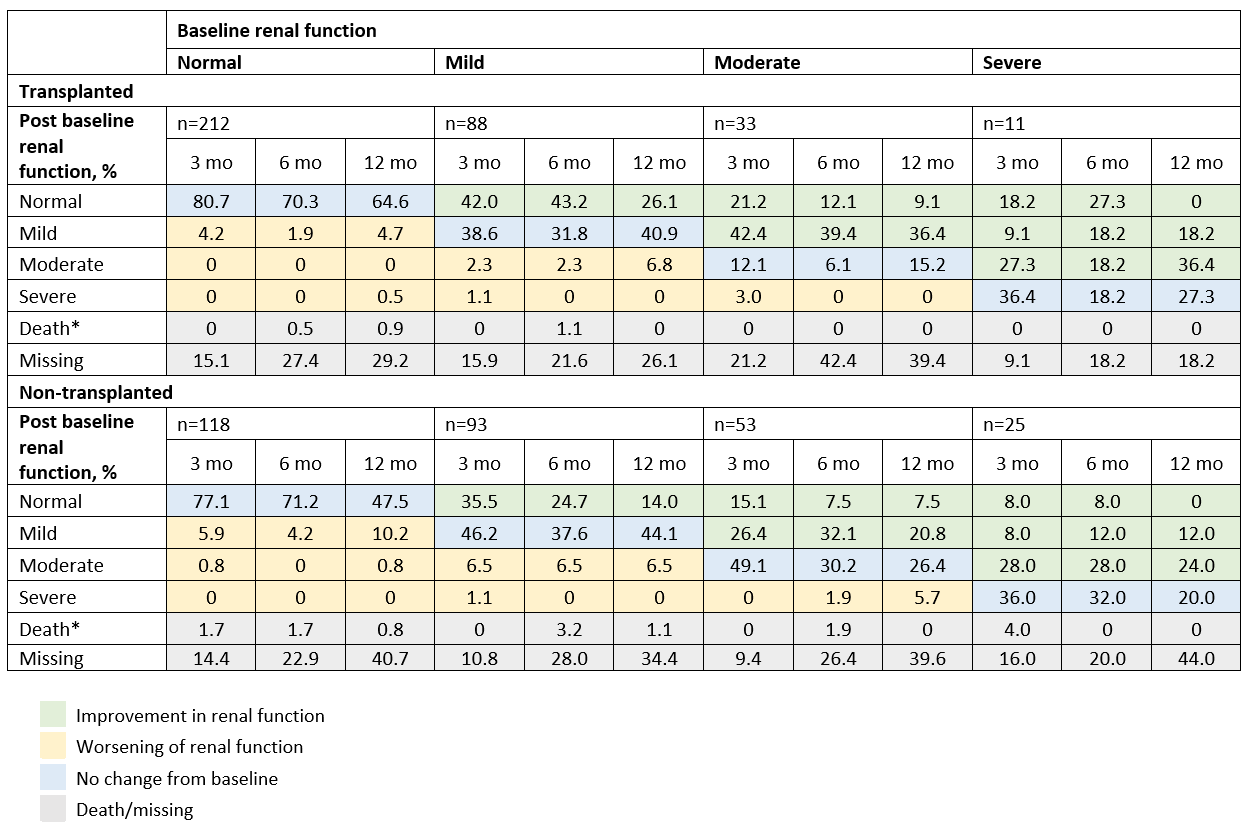


## **Figure S2. Unadjusted progression-free survival by baseline renal function for (a) transplant-eligible and (b) transplant-noneligible patients.**

Patients with first disease progression date earlier than their informed consent date (n = 2) were excluded from this analysis. Data cutoff date: August 4, 2021.


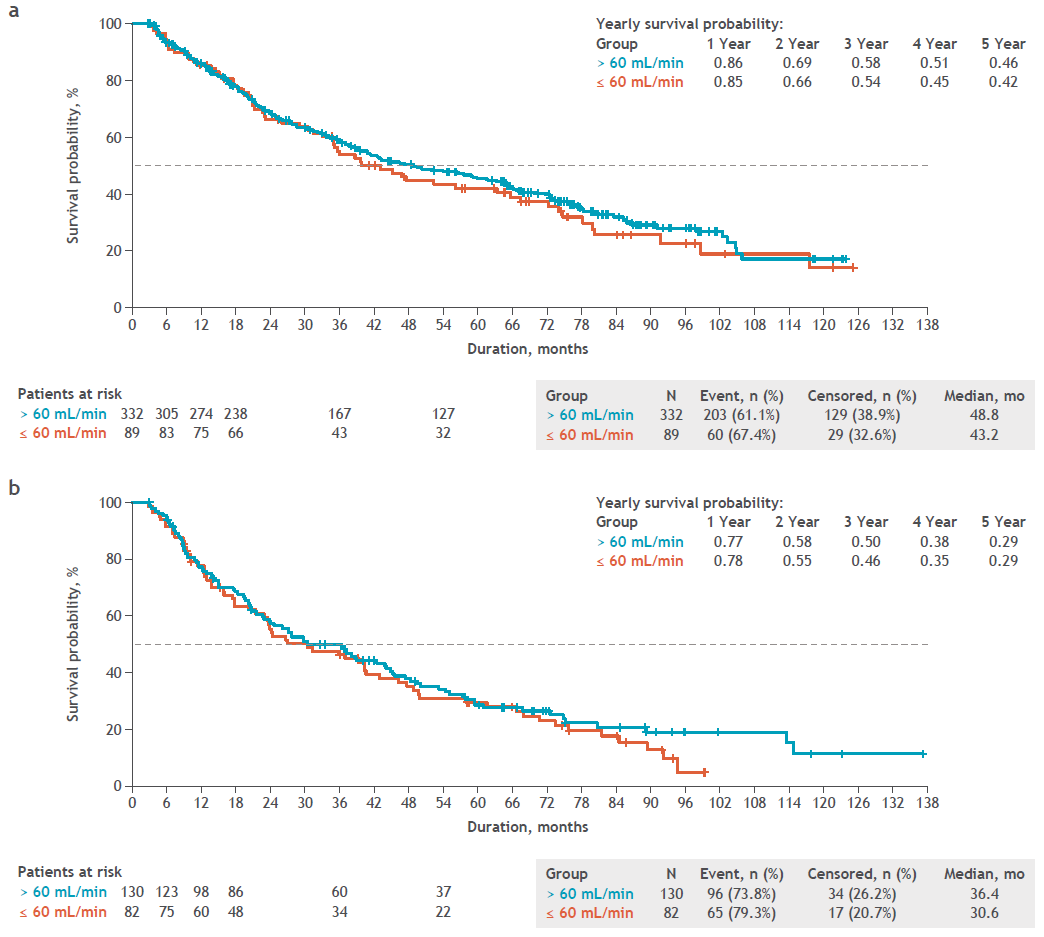

Supplement: Supplementary file 1 — Supplement [file 41408_2024_1177_MOESM1_ESM.docx]
